# Measles viral load may reflect SSPE disease progression

**DOI:** 10.1186/1743-422X-3-49

**Published:** 2006-06-21

**Authors:** M Kühne Simmonds, DWG Brown, L Jin

**Affiliations:** 1 Virus Reference Department, Centre for Infections, Health Protection Agency, 61 Colindale Avenue, London NW9 5HT, UK

## Abstract

Subacute sclerosing panencephalitis (SSPE) is a rare, slowly progressive neurological disorder caused by the persistent infection with measles virus (MV). Despite much research into SSPE, its pathology remains obscure. We examined autopsy tissues of eight SSPE patients by real time quantitative PCR, immunohistochemistry and immunoblotting to determine viral load. MV N, M and H gene RNA could be detected in the central nervous system (CNS) of all patients and in two non-CNS tissues of one patient. The viral burden between patients differed up to four-fold by quantitative PCR and corresponded with detection of MV protein. The level of both viral RNA and antigen in the brain may correlate with disease progression.

## Background

Subacute sclerosing panencephalitis (SSPE) is a rare, slowly progressive neurological disorder of children and young adults, caused by the persistent infection with measles virus (MV). After acute infection with MV, typically at a very young age, the patients appear to recover normally. Following a period of asymptomatic persistence, usually 3 to 10 years, neural dysfunctions develop and progress over months to years, resulting eventually in coma and death. Although much effort has gone into researching SSPE, its pathology remains largely obscure. The progression of SSPE greatly differs in individual patients [1]. What causes a rapid deterioration within months or slow progression over several years (4+) remains an unresolved question.

Viral load has been correlated to disease development, progression and outcome in several infections, including HIV, SIV, cytomegalovirus [2,3] and Epstein-Barr virus [4]. Occurrence of cognitive impairment in HIV infected patients and development of SIV encephalitis in macaques was linked to the level of viral RNA in the cerebrospinal fluid (CSF). In contrast, CNS disease did not correlate with plasma viral load in either HIV or SIV [5-7]. No studies have been carried out so far trying to relate the progression of SSPE with viral load. Due to the rarity of the condition and the invasiveness of removing biopsy samples, obtaining information on viral load during the course of disease is problematic. However, the use of autopsy samples may provide some insight into this relationship.

As MV can usually be detected in brain tissue from biopsy or autopsy by RT-PCR and/or immunohistochemistry, it is thought to persist and spread within the CNS. Although the brain appears to be the main location of persistent MV in SSPE, it is not entirely clear if this is also the site of persistence and the only target during disease. Viral antigen or RNA have been detected outside the CNS in SSPE cases [8-10]. To address the question of MV persistence outside the CNS in SSPE, we included available peripheral tissues of two SSPE patients in our analysis.

This study was carried out to determine whether differences in viral load caused diversity of disease progression. Autopsy tissues of brain and non-brain origin of eight confirmed cases of SSPE were analysed by quantitative PCR to determine amounts of the MV RNAs encoding the nucleoprotein (N), matrix protein (M) and haemagglutinin (H) and by immunological techniques to detect the N protein.

## Results

### Patients and samples

Autopsy or biopsy tissues of eight SSPE patients were analysed, details are shown in Table 1. All cases have been clinically and serologically confirmed as SSPE and MV RNA could be detected by RT-PCR in brain samples. Non-CNS tissues were available in two cases, liver, spleen and kidney of patient UK111, and liver, spleen, heart, lymph node, thymus, appendix and terminal ileum of UK98. All patients have been described previously [9,11].

**Table 1:** Samples.

Case	Gender	Available samples	Age at disease onset	Duration of persistence (acute MV to SSPE onset)	Duration of disease (SSPE onset to death)
UK111	F	Occipital brain	15 years	9–15 years	4 months
		Heart			
		Lung			
		Liver			
		Spleen			
UK98	M	Frontal brain	10 years	5–8 years	60 months
		Parietal brain			
		Occipital brain			
		Temporal brain			
		Cerebellum			
		Lymph node			
		Thymus			
		Heart			
		Lung			
		Spleen			
		Liver			
		Kidney			
		Tonsil			
		Appendix			
		Terminal ileum			
UK585	F	Frontal brain	24 years	19 years	18 months
		Temporal brain			
UK83	M	Brain	12 years	11 years	17 months
UK85	M	Brain	15 years	11 years	6 months
UK88	M	Brain	17 years	12 years	3 months
UK86	F	Brain	10 years	5 years^1^	34 months
UK87	M	Brain	7 years	5 years^1^	35 months

^1^Biopsy tissues were taken at disease onset.

### Detection of MV RNA

**Figure 1:**
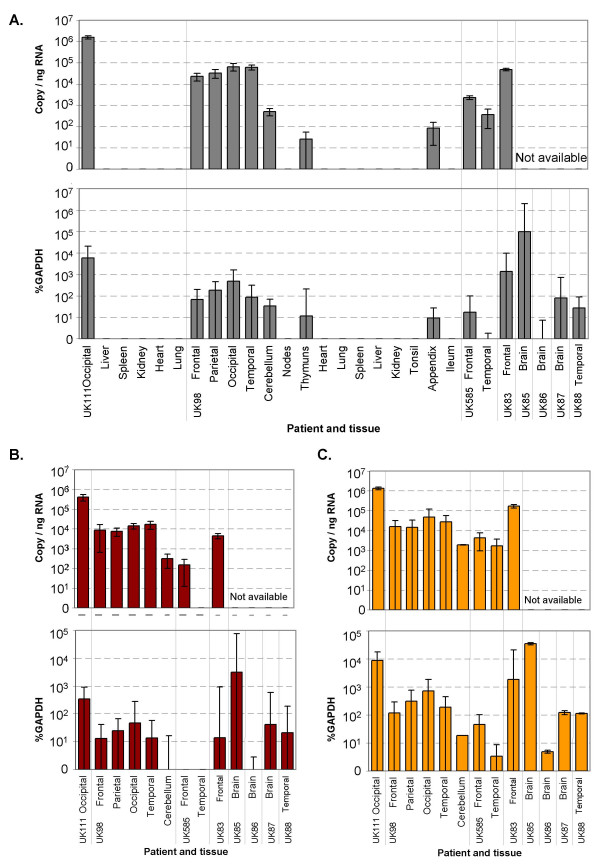
Detection of MV RNA by quantitative PCR in SSPE tissue samples. N (A), M (B) and H (C) RNA are shown and expressed as copy per ng total RNA and as percentage of detected GAPDH RNA. Values <1 were converted to 1 before logarithmic transformation. The results of one experiment are shown, representative for two with M an H genes and 3 with N gene. The error bars indicate 95% confidence intervals of duplicate samples.

MV N gene RNA was detected by quantitative real time PCR (qPCR) in all available CNS tissues from eight SSPE patients and in two non-CNS tissues of one patient. The concentration of MV N gene RNA in CNS tissues ranged from almost 2 × 10^6 ^copies of N RNA per ng total RNA in occipital brain patient UK111 to 4 × 10^2 ^copies/ng RNA in temporal brain of case UK585, respectively. In addition to relating copy numbers to the amount of total RNA, they were standardised as a function of GAPDH mRNA. GAPDH (glyceraldehyde-3-phosphate-dehydrogenase) is a ubiquitously expressed message, which is frequently used to standardise across tissues. Although expression of GAPDH may not be uniform across all tissue types, this method was found acceptable in a comparison of nine CNS and non-CNS tissues from three SSPE patients. The maximum fold change between CNS tissues was <8-fold, between CNS and non-CNS tissues <11-fold (Table 3). Expressed as units MV RNA per units GAPDH mRNA (%GAPDH) MV N RNA copy numbers in the CNS of the eight patients ranged from 6 × 10^3 ^%GAPDH in patient UK111 to <1% GAPDH in patient UK585. The only positive non-CNS tissues were thymus and appendix from patient UK98. In the thymus sample approximately 25 copies/ng RNA or 12% GAPDH were detected, in appendix 85 copies/ng RNA or 10% GAPDH. Results are shown in Figure 1 as copies per ng total RNA and as percentage of GAPDH RNA, except for samples from patients UK85, UK88, UK86 and UK87. These results are only shown as %GAPDH, as RNA was not concentrated enough to be quantified. Where both normalisation methods were available, similar relative results were obtained with both.

MV N RNA positive samples were also used for detection of MV M and H gene RNA. The H gene PCR lead to very similar results as the N gene PCR, both in copy numbers and %GAPDH. Occipital brain tissue of UK111 contained just under 2 × 10^6 ^copies of H RNA per ng total RNA. Again the weakest positive CNS sample was temporal brain of UK585 with just under 2 × 10^3 ^copies/ng RNA. This is approximately 9 × 10^3 ^%GAPDH and 3% GAPDH, respectively. The M gene signal was approximately ten-fold weaker, but the different cases were similar in relation to each other as with the N and H genes. Occipital brain of UK111 contained 4 × 10^5 ^copies M gene/ng RNA or 3 × 10^2 ^%GAPDH. This gene could not be detected in temporal brain tissue of patient UK585. In PCRs with MV N, M and H gene targets, occipital brain of UK111 was the strongest positive when looking at copies/ng RNA, while the brain of UK85 contained most MV RNA when expressed as a function of GAPDH. The efficiency of all three PCR reactions was found to be similar when titrations of p(+)MV, a MV genome containing plasmid, were used as template (data not shown), which indicates that the numbers obtained with the different PCRs can be compared. The M and H gene results confirm the relative findings with the N gene. All quantities are illustrated in Figure 1.

### Detection of MV protein

**Figure 2:**
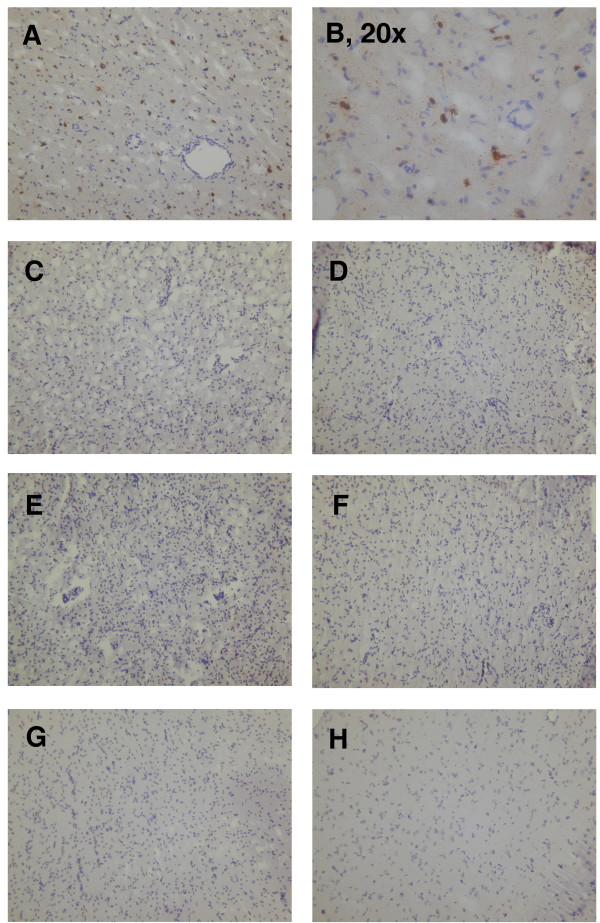
Detection of MV N protein by immunohistology. CNS tissue samples of three patients were analysed, and non-CNS tissues of two of them. The following sections are shown here. (A, B) UK111, brain. (C) UK98, frontal lobe. (D) UK98, parietal lobe. (E) UK98, occipital lobe. (F) UK98, temporal lobe. (G) UK585, frontal lobe. (H) UK585, temporal lobe. Original magnification 10 ×, unless otherwise indicated.

To determine whether the detected viral RNA is actively replicating, immunological methods were used to visualise MV protein in tissues from patients UK111, UK98 and UK585. In occipital brain tissue of UK111, a large number of MV N protein positive cells could be detected by immunohistochemistry, as seen in Figure 2. This staining of protein was not observed when an antibody against rubella virus was used. No MV N specific staining was located in any of the brain tissues of UK98 and UK585. There was no evidence of MV protein in any of the non-CNS tissues.

**Figure 3:**
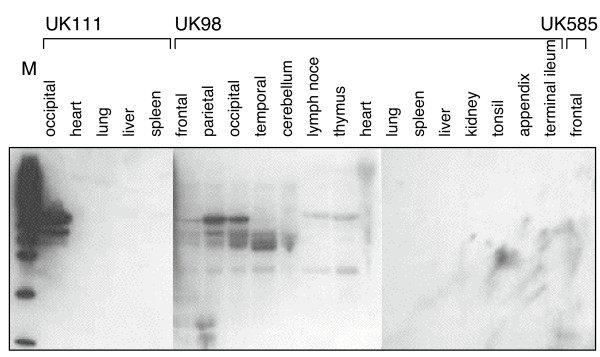
Detection of MV N protein in tissue samples by immunoblotting. Tissues were homogenised, lysed and protein denatured, before separating equal amounts of total protein by SDS-PAGE, Western blot analysis with a MV N specific antibody and chemiluminescent detection. M: weight marker.

To detect MV protein with higher sensitivity, tissue lysates were analysed by immunoblotting (Figure 3). In agreement with the findings using immunohistochemistry, a strong signal could be observed at 58 kDa in occipital brain of UK111. Additionally, N protein was also detected in the parietal and occipital brain tissues of UK98, and weakly in the frontal and temporal brain tissues of the same patient. A previously reported 45 kDa degradation product of N protein could also be seen in all samples with the 58 kDa band [12]. A weak band at 45 kDa was present in the lane containing the sample derived from cerebellum of UK 98, which may indicate low level of infection. No MV N protein could be detected in the frontal brain of UK585 or any of the non-CNS samples. It was essential not to remove cell debris by centrifugation after homogenisation and lysis, as this step lead to loss of bands in all samples from patient UK98 and reduced the band intensity of the strong band from brain tissue of UK111. This suggests that N protein aggregates and may bind to MV RNA or cellular RNA. These aggregates are large enough to be removed by centrifugation at 11,300 × g for 5 min.

### Disease progression, viral burden and MV M gene expression

**Figure 4:**
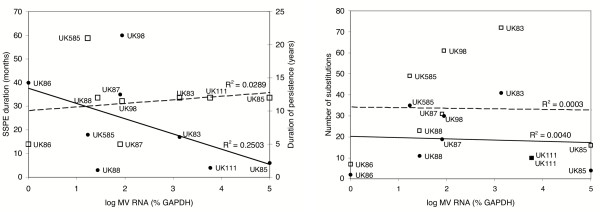
Relationship between viral load, disease duration and M gene substitutions. Linear correlation of log_10 _of viral load with (A) disease duration (●) and persistence from initial infection to sample date (□) or (B) number of amino acid (●) and nucleotide (□) substitutions in the MV M gene. R^2 ^values are indicated for each correlation. For patients UK98 and UK111, where length of persistence is not precisely known, an average was calculated from shortest and longest possible duration of persistence.

A weak inverse correlation between viral loads measured by qPCR and SSPE disease duration was detected. Quantities of viral RNA were compared to durations of SSPE and of infection from primary MV acquisition to sample date. Results are shown in Figure 4. While there was no correlation between quantity of MV RNA and total duration of infection, viral burden and SSPE duration may be linked. In patients where high load of viral RNA was measured and MV protein detected (UK111 and UK85), SSPE progressed rapidly and the patients died within four and six months of disease onset, respectively. The high viral burden may have accelerated the course of disease. Two subjects did not exhibit this tendency. In patient UK98, with intermediate viral load, disease progressed extremely slowly over 60 months. In UK88 on the other hand, very low levels of infection were detected but the patient died within three months of disease onset.

To investigate a possible link between virus integrity and viral spread, MV loads detected here were compared with the extent of MV M gene and protein mutation reported previously [11]. In cases UK111 and UK85 the MV M proteins contained few substitutions and a high virus load of 6 × 10^3 ^and 1 × 10^5 ^%GAPDH, respectively, was detected. The M gene of virus in case UK585, on the other hand, was strongly mutated and low virus levels of <1% GAPDH were measured. In case UK83, however, viral load was high (1 × 10^3 ^%GAPDH), despite presence of virus with a highly mutated M protein.

By Spearman's ranked correlation, no statistically significant links were found between viral load, duration of disease or infection and number of total or expressed mutations in the M gene of the isolated viruses.

## Discussion

Great variation in levels of MV RNA and protein were detected in different SSPE patients, while findings with quantitative PCR and immunological methods are in agreement. In the occipital brain of UK111, 2 × 10^6 ^copies of MV N RNA could be detected per ng total RNA, approximately 100 times more than in the brain samples of UK98. The brain of UK111 was the only analysed tissue positive by immunohistology and was a much stronger positive by immunoblotting than specimens from UK98. In the brain of UK585, which was negative by immunoblotting, only 4 × 10^2 ^to 2 × 10^3 ^copies could be detected. Thymus and appendix of UK98 were the only non-CNS samples positive for the N gene by real time PCR. GAPDH normalisation of these samples may be less reliable due to greater heterogeneity of tissue origin. However, taking into account exclusively the copy numbers per total RNA, they were weak positives, contained fewer than 10^2 ^copies per ng RNA.

A weak correlation between the amount of MV RNA and the duration of SSPE was observed. As some CNS samples contained relatively little MV RNA, high viral load alone did not seem to be responsible for death, but may have accelerated destruction of tissue. MV specific interferon-gamma release by host immune cells may determine disease progression [13]. It is feasible that release of high levels of interferon restricts viral growth and so reduces viral load and the speed of disease progression. Additionally, treatment with interferon or other antiviral agents during the course of SSPE may influence viral replication. Unfortunately, treatment history of the patients was not known and could therefore not be taken into account.

As the MV genome is encoded in RNA, PCR detection does not distinguish between genomic and mRNA. It is therefore not known if the identified virus is actively transcribed, but presence of MV protein indicates that the virus is transcribed. However, it is not clear whether a larger number of MV RNA copies indicate a greater number of infected cells or greater number of MV RNA copies per infected cell. Increased MV RNA levels per cell may indicate a more actively replicating virus, which may causes faster progressing disease. Alternatively, infection of a greater number of cells may lead to destruction of more tissue, which may also lead to accelerated disease.

Of patient UK98, tissues from five brain areas, frontal, parietal, occipital, temporal lobes and cerebellum, were available for analysis. Virus levels in the cerebrum – frontal, parietal, occipital and temporal lobes – were within a factor of 3.5, which may indicate that the virus did not favour any one particular area. Alternatively this may be an indication of very slow disease progression, as is the case in this patient, during which the virus had sufficient time to spread into all regions. In contrast, infection of the cerebellum was 30 to 100 times lower. The cerebellum is a very extensively connected part of the brain, which integrates information from the cerebrum as well as the periphery. Both efferent and afferent pathways link it to the cerebrum. Lack of axonal connection does therefore not cause this lower level of infection. It may rather be the different cell types present within this organ. The cerebellar neurons, the granule cells, may be less susceptible to MV infection.

The detection of MV RNA in non-CNS tissues was very low, which indicated that these tissues were not reservoirs of infection. The detected RNA may have derived from infiltrating lymphocytes that were infected within the CNS. Peripheral lymphocytes of individuals with SSPE have been shown to contain MV [14]. Alternatively, the source of MV sequences may have been contamination during autopsy. No information was available on the exact autopsy procedures, contamination could therefore not be ruled out.

In summary, the amount of MV protein and RNA differs greatly in different cases of SSPE, which may influence progression of the disease. Further studies of greater numbers of cases are required to confirm these findings.

## Materials and methods

### Real time PCR

Tissues were homogenised and total RNA extracted with RNeasy Mini kit (Qiagen, UK). RNA was treated with DNase to avoid amplification of genomic DNA. The concentration of extracted RNA was determined by spectrophotometry and 100 ng RNA reverse transcribed using random primers. PCR primers were designed using Primer3 software [15] to yield a 192, 223, 206, 238, 234 and 218 base pair amplification fragment of MV N, M or H gene, human GAPDH, beta-actin and 18S rRNA, respectively, and, where possible, read across intron/exon junctions to avoid amplification of genomic DNA (Table 2). PCR was performed in a reaction containing 5 μl RT product, 12.5 μl Platinum^® ^SYBR^® ^Green qPCR SuperMix UDG (Invitrogen, UK), 0.2 μM of each primer and 6.5 μl nuclease free water, using a Rotor-Gene 3000 (Corbett Research, UK) with the following cycling conditions. Hold at 50°C for 2 min, hold at 95°C for 3 min, followed by 45 cycles of 95°C for 15 sec, 55°C for 30 sec, 72°C for 30 sec. Data was acquired at 72°C during each cycle with excitation wavelength of 470 nm and detection at 585 nm. Amplification was followed by melt analysis from 50–90°C. All samples were run as duplicates and analysed using Rotor-Gene 6.0.7 software. Integrity of all samples was demonstrated by amplification of GAPDH mRNA. DNA and RNA standards were included in each run and each reaction was performed in duplicate. For verification of correct amplification, PCR products were run on agarose gel and sequenced.

**Table 2:** Primer sequences used for the amplification of MV and housekeeping genes with amplicon length.

Gene (GenBank accession number)	Primer sequence 5'-3'	Expected amplicon length
MV N	CAA GAC CCT GAG GGA TTC AA GTT CCT CAC CAC ATC CAA CC	192 bp
MV M	TGT TAC CCG CTG ATG GGT AT TTC TGG CTG TCA TTG TGA GG (M3'-1R, Jin 2002)	223 bp
MV H	ATC AGG GAT CAG GGA AAG GT CAT TCG CAA CTT GTC ATC TG (Mh4, Jin 1998)	206 bp
GAPDH (NM_002046)	GAG TCA ACG GAT TTG GTC GT TTG ATT TTG GAG GGA TCT CG	238 bp
beta-actin (NM_001101)	GGA CTT CGA GCA AGA GAT GG AGC ACT GTG TTG GGC GTA CAG	234 bp
18S rRNA (X03205)	CAT GGC CGT TCT TAG TTG GT CGC TGA GCC AGT CAG TGT AG	218 bp

GAPDH = glyceraldehydes-3-phosphate dehydrogenase, bp = base pairs

**Table 3:** Expression of housekeeping genes in tissues of SSPE patients.

Patient	Tissue	GAPDH (10^4 ^copy eqivalents/5 ng RNA)	Beta-actin (10^4 ^copy eqivalents/5 ng RNA)	18S rRNA (10^4 ^copy eqivalents/5 ng RNA)
UK98	Frontal brain	1.95	3.28	70.16
	Parietal brain	0.69	1.82	25.24
	Occipital brain	1.75	1.07	51.19
	Temporal brain	4.93	6.41	84.17
	Thymus	3.43	2.48	0.20
	Appendix	7.09	3.38	0.74
	Ileum	1.54	1.26	1.07
				
UK111	Occipital brain	1.46	3.51	83.00
				
UK585	Frontal brain	0.90	2.63	87.17

### Real time PCR controls

DNA and RNA standards were used for qPCR and RT-qPCR, respectively. The concentration of a plasmid containing the entire MV genome, p(+)MV (kindly provided by M. Billeter, University of Zürich), was determined by spectrophotometry. A ten-fold dilution series was made and used as DNA standard. From this plasmid, the whole N, M and H genes were amplified by PCR and cloned into pGEM (Promega, UK). The pGEM plasmid was linearised with SpeI and the MV fragments transcribed in vitro using the T7 Riboprobe System (Promega, UK) according to the manufacturer's instructions. The resulting RNA was purified and the concentration determined by spectrophotometry. Ten-fold dilution series were made and included in the RT reactions as RNA standards.

In order to compare results from different tissues and correct for use of different numbers of cells in each sample, two normalisation methods were employed. First, the same concentration of total isolated RNA was used for each RT-PCR. This lead to results expressed as copies of MV RNA per ng total RNA input. Alternatively, a housekeeping gene was employed to compare RNA levels between different samples. To find an appropriate gene to normalise sample input, 5 ng RNA from nine samples were used for qPCR with primers against all three housekeeping genes, beta-actin, GAPDH and 18S rRNA. Results are shown in Table 3. Both beta-actin and GAPDH were found to be suitable for normalisation, GAPDH was used in subsequent experiments. Results are expressed as units MV RNA/units GAPDH mRNA × 100 = amount MV RNA in %GAPDH.

### Immunohistochemistry

Formalin fixed, paraffin embedded tissues were analysed by immunohistochemistry. The method used by McQuaid et al. [16] was modified as follows. Formalin fixed, paraffin embedded 7 μm thick tissue sections on silane-coated slides were hydrated in xylene and alcohol. Endogenous peroxidase activity was blocked by 10 min incubation in 3% H_2_O_2 _in methanol. Tissues were washed in water and blocked in blocking solution (1:20 dilution of normal rabbit serum in PBS-T (0.1% Tween-20 in PBS)) for 10 min. Sections were incubated with a monoclonal mouse antibodies against the N protein (clone 7C11, [17]) at a dilution of 1:2000 in blocking solution overnight at 4°C, washed in PBS-T and incubated with a biotin conjugated secondary antibody (Dako, UK) at 1:400 in blocking solution for 1 hr. After further washing, streptavidin coupled HRP (Dako, UK) was added for 30 min, tissues washed and incubated with DAB, followed by haematoxylin counterstaining.

### Immunoblotting

Tissues were homogenised in lysis buffer (50 mM Tris, 150 mM NaCl, 2 mM EDTA, 2 mM EGTA, 1% Triton X-100 and added protease inhibitors) and protein concentrations of whole lysates, without removal of cell debris, determined by spectrophotometry. Equal amounts of total protein of each sample were analysed by SDS-PAGE and immunoblotting. The 7C11 MV N specific antibody was used to probe the blots, followed by detection with an anti-mouse-HRP antibody (Dako, UK) and ECL plus reagents (Amersham Bioscience, UK).

## Authors' contributions

MKS conceived the study, carried out the laboratory work and drafted the manuscript. JL and DWGB participated in the design of the study and the revision of this manuscript. All authors read and approved the final manuscript.
